# Advancements in the interventional therapy and nursing care on deep vein thrombosis in the lower extremities

**DOI:** 10.3389/fmed.2024.1420012

**Published:** 2024-07-26

**Authors:** Chun-yi Jia, Dan-dan Dai, Xin-yuan Bi, Xia Zhang, Yi-ning Wang

**Affiliations:** ^1^Department of Intervention, The Second Affiliated Hospital of Mudanjiang Medical University, Mudanjiang, China; ^2^Department of Nursing Care, The Second Affiliated Hospital of Mudanjiang Medical University, Mudanjiang, China; ^3^Department of Vascular Surgery, The Second Affiliated Hospital of Mudanjiang Medical University, Mudanjiang, China; ^4^Department of Cardiology, The Second Affiliated Hospital of Mudanjiang Medical University, Mudanjiang, China

**Keywords:** deep vein thrombosis, lower extremity, interventional therapy, anticoagulants, nursing care

## Abstract

This review examines recent advancements in interventional treatments and nursing care for lower extremity deep vein thrombosis (DVT), highlighting significant innovations and their clinical applications. It discusses the transition to novel anticoagulants such as Direct Oral Anticoagulants, which offer a safer profile and simplified management compared to traditional therapies. Mechanical interventions, including balloon angioplasty and venous stenting, are detailed for their roles in improving immediate and long-term vascular function in acute DVT cases. Furthermore, the use of image-guided techniques is presented as essential for enhancing the accuracy and safety of DVT interventions. Additionally, this study outlines advances in nursing care strategies, emphasizing comprehensive preoperative and postoperative evaluations to optimize patient outcomes. These evaluations facilitate tailored treatment plans, crucial for managing the complex needs of DVT patients. Long-term care strategies are also discussed, with a focus on patient education to ensure adherence to treatment protocols and to prevent recurrence. The synthesis aims to inform healthcare professionals about cutting-edge practices in DVT management, promoting a deeper understanding of how these advancements can be integrated into clinical practice. It also underscores the necessity for ongoing research to address challenges such as cost-effectiveness and patient compliance, ensuring that future treatments are both accessible and effective.

## 1 Introduction

Deep vein thrombosis (DVT) represents a significant medical condition characterized by the formation of blood clots within the major veins of the lower extremities (1, 2). This condition not only induces pain, swelling, and limited mobility in the affected limb but also poses a severe risk of life-threatening pulmonary embolisms should a clot dislodge and migrate to the lungs (1, 2). Given its prevalence and the severe complications associated with it, DVT is a critical public health issue demanding effective strategies for its prevention, diagnosis, and management (3).

Traditionally, the management of DVT has centered around anticoagulant therapy, the use of compression stockings, and occasionally, surgical interventions (4). While these treatments have proven beneficial, they come with notable limitations (5). Anticoagulants, for example, while effective in preventing clot progression and new clot formation, require rigorous monitoring to mitigate the risk of severe bleeding complications. Compression stockings are beneficial for reducing thrombus formation and edema but often face challenges with patient compliance (6). Furthermore, these conventional approaches do not fully address the recurrence of the condition or the long-term complications associated with DVT (6).

In response to these challenges, there has been a marked shift towards more innovative interventional treatments and care protocols in recent years (7). Advanced treatment modalities, including novel anticoagulants, mechanical thrombectomy, and image-guided minimally invasive procedures, have been developed (8, 9). These innovations offer enhanced efficacy and safety, showing particular promise in the management of severe or complex DVT cases.

The objective of this mini review is to conduct a thorough evaluation and synthesis of the current scientific and clinical advancements in the interventional treatment and management of DVT. This mini review will scrutinize the latest research findings and clinical trials, aiming to elucidate how these innovative approaches have refined patient outcomes. Moreover, it explores prospective research avenues and potential advancements in technology that could further revolutionize the treatment landscape of DVT.

This review is crucial for healthcare professionals as it not only highlights contemporary treatment methodologies but also lays down a comprehensive framework for future investigations. Such rigorous examination is essential for advancing clinical practice and enhancing patient care in the realm of DVT.

## 2 Recent advances in interventional therapy of DVT in lower extremities

### 2.1 Pharmacological interventions

Pharmacological interventions play a pivotal role in the management of DVT, with significant advancements in both drug types and therapeutic strategies in recent years. Modern pharmacotherapy primarily involves anticoagulants and thrombolytics, which have substantially evolved to improve both efficacy and safety for patients (10–18) (Table 1).

**Table 1:** Interventional therapy in patients with DVT in the lower extremities.

Reference	Type of study	Treatment	Key findings
Schulman et al. (10)	Randomized Controlled Trial	Dabigatran vs. warfarin	A fixed dose of dabigatran is as effective and safe as warfarin and does not require laboratory monitoring.
Agnelli et al. (11)	Randomized Controlled Trial	Apixaban	A fixed-dose regimen of apixaban was noninferior to conventional therapy for acute VTE and resulted in significantly less bleeding.
Prins et al. (12)	Observational Study	Rivaroxaban	Rivaroxaban showed similar efficacy to standard therapy with a significantly lower rate of major bleeding.
Trca et al. (13)	Randomized Controlled Trial	Pentasaccharide medication	Potential protective effects of pentasaccharides against DVT development may be expected.
Okuda et al. (14)	Randomized Controlled Trial	Heparin and intermittent pneumatic compression	Prevention of DVT post-laparoscopic cholecystectomy.
Verhaeghe et al. (15)	Pilot Study	Systemic rt-PA	Efficacy and safety in treating DVT of lower extremities.
Fronas et al. (16)	Observational Study	Rivaroxaban	Feasibility of rivaroxaban in deferred workup of suspected DVT.
Napolitano et al. (17)	Randomized Controlled Trial	Low molecular weight heparin	Six months of LMWH lowers thrombosis recurrence risk; extending treatment in those with residual vein thrombosis does not further reduce VTE.
Chang et al. (18)	Observational Study	Alteplase	Intraclot injection of alteplase directly into the thrombus is an alternative to continuous infusion, reducing systemic exposure to thrombolytics.
Meng et al. (19)	Randomized Control Trial	Stenting post-thrombolysis	Stenting iliac vein obstructions after CDT for lower extremity DVT may improve deep vein patency, enhancing efficacy and quality of life.
Snyder et al. (20)	Randomized Control Trial	Mechanical compression device and aspirin	Prevention efficacy post-total knee arthroplasty.
Li et al. (21)	Retrospective Study	Balloon-assisted CDT	Balloon-assisted CDT is a superior, safer treatment for acute DVT, reducing medication, procedure time, and increasing patency without raising PE risk.
Sukovatykh et al. (22)	Observational Study	Venous stenting	Stenting in acute period of total and subtotal DVT.
Comerota et al. (23)	Retrospective Study	CDT	Improved health-related quality of life in iliofemoral DVT.
Davidson et al. (24)	Observational Study	Color Doppler sonography	Screening for DVT in asymptomatic postoperative patients.
Vedantham et al. (25)	Consensus Report	Endovascular treatment	Reporting standards for endovascular treatment of DVT.
Kim et al. (26)	Observational Study	CT imaging	DVT density’s clinical value for predicting pulmonary thromboembolism.
Mani et al. (27)	Randomized Control Trial	MRV and gadofosveset	MRV with gadofosveset trisodium reliably estimates thrombus volume in DVT in a multicenter setting.

DVT, deep vein thrombosis; VTE, venous thromboembolism; LMWH, low molecular weight heparin; CDT, catheter-directed thrombolysis; MRV, magnetic resonance venography.

#### 2.1.1 Anticoagulation therapy

The advent of direct oral anticoagulants (DOACs), including rivaroxaban, apixaban, and dabigatran, marks a significant shift from traditional warfarin therapy (10–18) (Table 1). DOACs offer advantages such as reduced need for monitoring, fewer dietary and drug interactions, and a more stable therapeutic profile. Not requiring frequent INR monitoring, these anticoagulants simplify outpatient management and enhance patient adherence.

Clinical trials such as EINSTEIN, RE-COVER, and AMPLIFY have demonstrated the efficacy of DOACs in treating DVT with a notably lower risk of bleeding compared to traditional anticoagulants. These studies underline the role of DOACs in setting new standards for anticoagulation therapy (10–18).

Alongside established therapies, the development of anti-XI anticoagulants marks an innovative frontier in thromboprophylaxis (28). These agents specifically inhibit Factor XI, offering a targeted approach to reducing thrombosis risk while potentially minimizing bleeding complications—a common limitation of traditional anticoagulants (28, 29). Emerging clinical trials have begun to explore the efficacy and safety profiles of these anticoagulants, indicating their promise for safer therapeutic regimens in populations at high risk of bleeding (30). The introduction of anti-XI agents underscores the dynamic evolution of anticoagulant therapy, aiming to balance efficacy and safety more effectively (31).

#### 2.1.2 Thrombolytic therapy

Tissue plasminogen activator, such as alteplase, continues to be extensively utilized for acute DVT management, effectively dissolving clots and restoring venous flow (14, 15) (Table 1). Its application is crucial in managing severe thrombotic events where rapid resolution is necessary.

The ATTRACT trial, among others, has explored the role of thrombolytics in the early intervention of DVT to prevent post-thrombotic syndrome and chronic venous insufficiency. While highlighting the benefits in symptom reduction, these studies also stress the importance of careful patient selection to mitigate bleeding risks.

### 2.2 Mechanical interventional treatments

The development of mechanical interventional techniques has introduced novel therapeutic possibilities for DVT, especially beneficial for patients who are not ideal candidates for conventional anticoagulation (19, 20) (Table 1).

#### 2.2.1 Balloon angioplasty

This technique involves the insertion of a catheter with an attached balloon into the thrombosed vein (21). Upon inflation, the balloon physically expands the vein, displacing the clot and restoring blood flow. This approach is particularly effective in acute cases where rapid amelioration of symptoms is critical (21) (Table 1). Balloon angioplasty is typically reserved for critical cases requiring rapid restoration of venous flow due to significant obstruction (21). This intervention is particularly crucial in severe phlegmasia dolens, where there is an immediate risk of limb ischemia.

#### 2.2.2 Venous stenting

Stenting involves the placement of a metallic mesh within the vein to maintain vascular patency and prevent future occlusions. It is particularly applicable in scenarios where venous stenosis persists post-thrombosis or when angioplasty alone does not suffice to restore normal blood flow (19, 22). The stent acts as a scaffold, ensuring long-term patency and improving overall venous return, which is crucial for reducing recurrence rates and improving long-term outcomes (19, 22). Stenting is primarily recommended for managing chronic venous insufficiency, particularly when compressive symptoms continue to persist despite the application of less invasive treatments (32).

Recent clinical data affirm that mechanical interventions like balloon angioplasty and stenting significantly enhance symptom management and quality of life (19, 22) (Table 1). However, the sustainability of treatment effects and the potential for long-term complications necessitate further research and careful patient follow-up.

### 2.3 Image-guided interventional strategies

Image-guided techniques have revolutionized the precision and safety of interventions for managing DVT, proving essential in both diagnosis and therapeutic application.

#### 2.3.1 Ultrasound guidance

Color Doppler ultrasound is the frontline modality for diagnosing DVT, providing real-time, dynamic blood flow information. It is indispensable for guiding minimally invasive procedures such as catheter-directed thrombolysis, allowing for direct visualization of the thrombus and treatment effects (23–25) (Table 1).

Advancements in Ultrasound Technology: The field of ultrasound technology has seen significant advancements, notably in the development of portable, high-resolution devices (33). These improvements have expanded the reach of ultrasound beyond traditional clinical settings, facilitating its use in remote locations and potentially increasing patient access to timely diagnosis and management.

Future Directions in Ultrasound and Digital Health Integration: Looking forward, the integration of ultrasound technology with digital health applications holds promising potential for enhancing patient management (34). Innovations on the horizon include wearable devices capable of continuous monitoring of vital parameters such as heart rate, oxygen saturation, and electrocardiographic signals (34). These devices could play a crucial role in early detection of complications, such as pulmonary embolism, and enhance preventive care. Furthermore, advancements are expected in the development of smart medication adherence tools that remind patients of dosage schedules, thereby improving compliance with treatment protocols (35).

These technological advancements are poised to transform the management of DVT, enhancing both the efficacy and safety of treatments and supporting proactive patient care through continuous monitoring and timely intervention.

#### 2.3.2 Advanced imaging techniques

Computed tomography (CT) or magnetic resonance imaging (MRI) offers comprehensive imaging capabilities that extend beyond the limitations of ultrasound, especially useful in complex cases where clots are located in anatomically challenging positions (26, 27). These modalities enable detailed visualization of the vascular anatomy, facilitating precise interventions such as stenting and targeted thrombolysis, ensuring interventions are performed with the highest accuracy (26, 27) (Table 1).

Specifically, the use of CT and MRI is highly restricted and generally recommended in specialized cases such as left iliac DVT in pregnant patients (26, 27). In these patients, MRI is preferred over CT due to the absence of ionizing radiation, which poses a risk to the developing fetus (27). This ensures that both mother and fetus are safeguarded while obtaining essential diagnostic information to guide further treatment.

The integration of image-guided technology not only enhances the effectiveness of therapeutic interventions but also minimizes procedural risks, thereby improving patient safety and treatment outcomes. As imaging technology advances, future interventions are expected to become even more refined, further elevating the standard of care for patients with DVT.

## 3 Recent advances in nursing strategies of DVT in lower extremities

### 3.1 Preoperative and postoperative care

Effective preoperative and postoperative care is crucial in optimizing outcomes and minimizing complications in the interventional treatment of lower extremity DVT. Advancements in medical technology have greatly enhanced the precision of nursing assessments, which now play a pivotal role in the planning and execution of treatment (Table 2).

**Table 2:** Nursing strategies in patients with DVT in the lower extremities.

Reference	Type of study	Treatment	Key findings
Kearon et al. (6)	Practice Guidelines	Antithrombotic therapy for VTE	Provided guidelines for antithrombotic therapy in VTE.
Heit et al. (36)	Retrospective Study	Not applicable	This underscores the need to identify at-risk patients and understand VTE to ensure effective prophylaxis.
CLOTS Trials Collaboration (37)	Randomized Controlled Trial	Thigh-length graduated compression stockings	Evaluated effectiveness in preventing DVT post-stroke.
Ho et al. (38)	Meta-analysis	Intermittent pneumatic compression	Prevented VTE in hospitalized patients.
Kakkos et al. (39)	Systematic Review	Combined intermittent pneumatic compression and pharmacological prophylaxis	Prevention of VTE.
Rawal (40)	Review Article	Not applicable	Discussed current issues in postoperative pain management.
Kahn et al. (41)	Observational Study	Not applicable	Analyzed determinants and time course of postthrombotic syndrome.
Wells et al. (42)	Diagnostic Study	Evaluation of D-dimer	Assessed the utility of D-dimer in diagnosing suspected DVT.
Pezold et al. (43)	Case Report	Percutaneous mechanical thrombectomy	Described thrombectomy in a pediatric patient with DVT.
Crişan et al. (44)	Descriptive Study	Online methods of patient education	Evaluated effectiveness of online education for DVT patients.

DVT, deep vein thrombosis; VTE, venous thromboembolism.

Nursing Assessments: Modern nursing practices involve comprehensive evaluations using standardized tools that assess hemorheology, limb measurements, and a patient’s overall physiological and psychological state. These evaluations help clinicians identify patient-specific risk factors and predict potential complications, allowing for the creation of tailored interventional treatment plans (6, 36). Furthermore, assessments extend to evaluating a patient’s understanding and preparedness for upcoming treatments, ensuring they are fully informed and ready both mentally and physically.

Preoperative Preparations: Key aspects of preoperative care include thorough health examinations to ascertain the patient’s baseline health status and detailed discussions to gauge their understanding and acceptance of the proposed interventions (37–39). Educating patients about what to expect during and after the procedure is critical. This education covers potential postoperative scenarios and adherence to prescribed self-care protocols.

Postoperative Management: Focuses on managing pain and overseeing wound care, essential for facilitating a swift recovery and optimizing the treatment’s success. Pain management strategies often encompass both pharmacological and non-pharmacological approaches, including physical therapy (40). Wound care involves regular monitoring and timely intervention if signs of infection arise, preventing further complications. Postoperative care also includes functional rehabilitation and ongoing psychological support, fostering a holistic recovery approach.

Implementing these refined care strategies can significantly enhance patient outcomes, reducing the risks and complications associated with the treatment of DVT (Table 2).

### 3.2 Long-term care and patient education

Managing lower extremity DVT extends beyond immediate medical intervention and encompasses long-term care and patient education, which are essential for the sustained well-being of patients (Table 2).

Home Care: After hospital discharge, home care becomes a critical aspect of a DVT patient’s routine, particularly during the recovery and maintenance phases. Patients need comprehensive instructions and continuous support for activities such as adhering to anticoagulation regimens and monitoring for adverse effects (41, 42). The ongoing anticoagulation is crucial for preventing thrombus reformation but carries an inherent risk of bleeding, necessitating patient education on managing these risks effectively (41, 42).

Lifestyle Modifications: Adjustments in daily living, such as maintaining an active lifestyle, achieving and sustaining an appropriate weight, and following a heart-healthy diet, are crucial (43). These modifications help mitigate the risk of recurrent DVT and enhance overall health.

Patient Education: This is an integral component of long-term management, empowering patients with knowledge about their condition, the importance of their treatment regimen, and the ability to recognize symptoms of potential complications (44). Education should not only provide disease and treatment-related information but also include practical advice on when and how to seek further medical assistance (44). Effective education transforms patients from passive recipients of healthcare into active participants in their health management, aiming to prevent recurrence and elevate their quality of life.

By enhancing these aspects of care, healthcare providers can ensure better management of DVT, promoting patient independence and improving outcomes in long-term disease management.

## 4 Nursing strategies for special populations in the treatment of DVT in lower extremities

Elderly Patients: Elderly individuals undergoing DVT intervention often present with multiple comorbidities, such as cardiovascular diseases and renal insufficiency, which can affect their response to anticoagulant therapy (45). The diminished drug metabolism typical in older age further elevates the risk of adverse reactions during treatment. Hence, nursing care for elderly patients should include meticulous monitoring of drug dosages and therapeutic responses. Care plans must also consider potential cognitive impairments and the availability of family support, tailoring education and support programs to meet their specific needs. This ensures that elderly patients receive care that is both effective and sensitive to their particular health status.

Pregnant Women: DVT treatment in pregnant women requires careful consideration of both maternal and fetal health due to the teratogenic risks associated with many standard anticoagulants (46–48). The selection and timing of therapeutic interventions must prioritize safety, avoiding any potential harm to the developing fetus. Physiological changes during pregnancy can alter the risk profile for thrombus formation and modify responses to treatment, necessitating vigilant monitoring and management (46–48). Collaborative efforts with obstetric specialists are essential to tailor interventions that safeguard the health of both mother and child, ensuring that treatment protocols are precisely followed.

Patients with Chronic Diseases: Individuals with chronic conditions such as cancer, diabetes, or cardiovascular disease may face additional challenges when undergoing DVT interventions (3, 49). These conditions can influence both the choice of treatment and the overall prognosis. For instance, cancer patients are particularly susceptible to increased thrombotic risk due to the malignancy and associated treatments like chemotherapy. Nursing care strategies must be adaptive to these risks, adjusting therapeutic approaches to effectively manage and minimize the likelihood of recurrence. It is also crucial for the care team to address the comprehensive needs of these patients, including nutritional support, effective pain management, and psychological care, to enhance their overall quality of life and treatment outcomes.

In summary, effective nursing care for special populations undergoing treatment for lower extremity DVT requires a detailed understanding of each individual’s unique medical and personal circumstances. By implementing personalized and meticulously planned care strategies, healthcare providers can ensure that interventions are not only safe and effective but also aligned with the specific needs and conditions of elderly patients, pregnant women, and those with chronic diseases. This approach promotes optimal health outcomes and patient satisfaction.

## 5 Challenges and future directions in the interventional treatment of DVT in lower extremities

Despite notable advancements in the management of lower extremity DVT through interventional treatments, several challenges persist that affect the adoption and efficacy of these approaches.

### 5.1 Technological limitations

Current interventional techniques such as thrombectomy and catheter-directed thrombolysis are effective but involve complex procedures that require a high degree of technical skill (3, 50, 51). The reliance on sophisticated medical equipment also limits the application of these treatments in resource-constrained settings (3, 50, 51). Addressing these technological constraints involves simplifying procedures and developing equipment that is both cost-effective and suitable for use in a broader range of healthcare environments.

### 5.2 Cost-effectiveness

The high costs associated with interventional treatments for DVT pose significant barriers to their widespread use (41, 49–53). These costs can be prohibitive for patients and healthcare systems, especially in lower-income areas. Future research must focus on conducting thorough cost-effectiveness analyses to identify economic strategies that can reduce expenses while maintaining or enhancing clinical outcomes. Exploring generic medication options, streamlined procedural techniques, and cost-sharing models could be viable approaches.

### 5.3 Patient acceptance

The acceptance of new or complex interventional treatments by patients is crucial for successful outcomes (41, 54). Challenges in patient education and the communication of treatment benefits and risks are significant (41, 54). Improving educational strategies to better inform patients about their options, the procedures involved, potential risks, and expected outcomes is essential. Enhanced communication efforts should aim to build trust and understanding, facilitating informed consent and engagement in treatment decisions.

### 5.4 Future research and innovations

Research in the coming years may concentrate on creating simpler, more affordable interventional techniques. There is a need to innovate in patient education and care coordination to adapt to the evolving landscape of healthcare delivery. Future developments could include (55–57):

Technology Advancements: Redesigning devices and simplifying interventional procedures to make them more accessible and easier to perform.

Digital Tools: Leveraging technology to enhance patient education, treatment transparency, and engagement through digital platforms that provide accessible information and support.

Interdisciplinary Approaches: Developing collaborative care models that integrate various healthcare professionals, including doctors, nurses, physical therapists, and social workers, to deliver holistic and coordinated care.

Such innovations are expected to optimize the management of DVT, significantly improve clinical outcomes, and broaden the application of interventional treatments, making them more accessible and effective across diverse patient populations and geographic locations.

## 6 Summary

A thorough evaluation of the recent developments in interventional treatments and nursing strategies for lower extremity DVT reveals several critical insights. Firstly, technological advancements in interventional methods have notably increased the efficiency and safety of treatments for DVT. The adoption of advanced anticoagulants, mechanical thrombectomy techniques, and image-guided minimally invasive surgeries has shown exceptional effectiveness in clinical settings, particularly in managing complex or high-risk DVT cases. Furthermore, enhancements in nursing strategies, including detailed assessments before and after surgery, effective pain management, and comprehensive long-term care coupled with patient education, have substantially improved both the rehabilitation outcomes and the overall quality of life for patients.

Despite these advancements, the need for continuous research is imperative. Future investigations should delve deeper into the capabilities of emerging technologies, especially focusing on their role in minimizing complications and fostering long-term wellness for patients. Moreover, there is a critical need to focus on increasing patient compliance with treatment regimens and improving the effectiveness of interdisciplinary collaborations to streamline and execute treatment protocols more efficiently. By addressing these areas, we can further refine DVT treatment processes and enhance the quality of care provided to patients, ultimately leading to significant improvements in their quality of life.

## Author contributions

C-yJ: Conceptualization, Data curation, Methodology, Resources, Validation, Visualization, Writing – original draft, Writing – review & editing. D-dD: Data curation, Methodology, Resources, Validation, Visualization, Writing – original draft, Writing – review & editing. X-yB: Conceptualization, Methodology, Resources, Validation, Visualization, Writing – original draft, Writing – review & editing. XZ: Resources, Validation, Visualization, Writing – original draft, Writing – review & editing. Y-nW: Conceptualization, Data curation, Investigation, Project administration, Resources, Supervision, Validation, Visualization, Writing – original draft, Writing – review & editing.
